# Right Heart Failure in Critical and Chronic Care: Current Concepts, Challenges and Mechanical Support Strategies

**DOI:** 10.3390/medsci13040210

**Published:** 2025-09-28

**Authors:** Debora Emanuela Torre, Carmelo Pirri

**Affiliations:** 1 Department of Cardiac Anesthesia and Intensive Care Unit, Cardiac Surgery, Ospedale dell’Angelo, 30174 Venice Mestre, Italy; 2 Department of Neurosciences, Institute of Human Anatomy, University of Padova, 35121 Padova, Italy; carmelo.pirri@unipd.it

**Keywords:** right heart failure, right ventricular failure, mechanical circulatory support, right ventricular assist device, veno-arterial ECMO, ECPELLA, echocardiography, biomarkers, critical care, advanced heart failure, RV dysfunction

## Abstract

Right heart failure (RHF) remains an under-recognized yet devastating condition in critically ill and chronic patients, frequently complicating cardiac surgery, pulmonary embolism, advanced heart failure, sepsis and left ventricular assist device (LVAD) implantation. Despite growing awareness, clinical decision making is still hampered by the complex pathophysiology, limitations in diagnosis and a fragmented therapeutic landscape. In recent years, progress in hemodynamic phenotyping, advanced echocardiographic and biomarker-based assessment, and the development of mechanical circulatory support (MCS) systems, including percutaneous and surgical right ventricle assist devices (RVAD), veno-arterial extracorporeal membrane oxygenation (V-A ECMO), Impella RP (right percutaneous) or BiPella (Impella CP/5.0/5.5 + Impella RP) has expanded the armamentarium for managing RHF. This review synthetizes current evidences on the anatomical, physiological and molecular underpinnings of RHF, delineates the distinction and continuum between acute and chronic forms and provides a comparative analysis of diagnostic tools and MCS strategies. By integrating mechanistic insights with emerging clinical frameworks, the review aims to support earlier recognition, tailored management and innovative therapeutic approaches for this high-risk population.

## 1. Introduction

Right heart failure (RHF) is a clinical syndrome resulting from dysfunction of the right-sided circulatory system, leading to elevated venous pressures and/or impaired blood delivery to the pulmonary circulation. It may arise from abnormalities affecting any component of the right heart circuit, which extends from the systemic venous system up to the pulmonary capillaries, thereby encompassing both systemic (the systemic veins up to the pulmonic valve) and pulmonary (the precapillary pulmonary circulation) domains [1]. Within this framework, the more specific concept of right ventricular failure (RVF) is often central. Hemodynamically, RVF is defined as the point at which cardiac output and systemic blood pressure decline despite increased right ventricular end-diastolic pressure (RVEDP) [2]. Predominant right-sided congestion was defined by an elevated right atrial pressure (RAP)/pulmonary artery occlusion pressure (PAOP or PCWP) ratio (typically > 0.63 and approaching or exceeding 1 in overt RHF), particularly when accompanied by RAP ≥ 15 mmHg, usually with a low cardiac index (CI) [3]. RHF occurs when the right ventricle fails to maintain adequate blood flow through the pulmonary circulation to ensure sufficient left ventricular filling. Reported in-hospital mortality ranges from 5% to 17% [4].

This may develop acutely in a previously healthy heart, for example following massive pulmonary embolism or right-sided myocardial infarction, or it may represent the decompensation of chronic RV dysfunction in the setting of long-standing cardiopulmonary disease. Management of RHF requires careful optimization of preload and reduction of afterload. Given the relatively low vascular tone of the pulmonary circulation, vasoactive drugs exert less effect on pulmonary vascular resistance than on systemic resistance. Therefore, treatment focuses on correcting reversible factors that exacerbate pulmonary vascular tone, employing selective pulmonary vasodilators at doses that avoid systemic hypotension or worsening hypoxemia. Systemic systolic arterial pressure should be maintained close to RV systolic pressure to preserve RV perfusion [5]. When these measures are insufficient, inotropic agents may be judiciously used to augment contractility and sustain cardiac output. In refractory cases, extracorporeal life support (ECLS) provides a valuable option, offering temporary circulatory assistance while the underlying etiology of RV failure is addressed. Advances in pulmonary vascular biology, novel classes of pulmonary vasodilators and the evolution of ECLS technologies have collectively expanded the therapeutic armamentarium and deepened the understanding of RV physiology [6]. This review was conducted using a structured narrative approach. PubMed/MEDLINE and Scopus were searched up to 2025 with controlled vocabulary and free-text terms related to RHF, pulmonary hypertension, cardiogenic shock and mechanical circulatory support. Additional references were identified through citation tracking. Non-English publications and conference abstracts without full text were excluded. Evidence was thematically synthesized across anatomy, pathophysiology, diagnostics and therapeutic strategies.

## 2. Relevant Sections

### 2.1. Right Ventricular Anatomy, Function and Interplay with the Pulmonary Circulation

**Figure 1:**
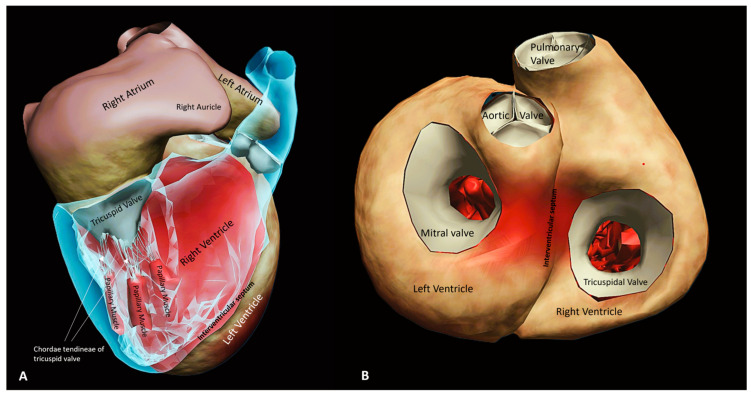
Right ventricular anatomy and geometry. (**A**) Right-sided cutaway showing the thin-walled, trabeculated RV with inlet (tricuspid annulus, leaflets, chordae, papillary muscles), trabecular apex and outflow tract (infundibulum) separated by the crista supraventricularis. (**B**) Basal short-axis view highlighting the crescentic RV wrapping the LV and the valve relationship (pulmonary anterior-superior, aortic central, tricuspid right/anterior, mitral left/posterior). The interventricular septum, as a shared wall, modulates ventricular interaction and contributes to the RV’s high compliance yet afterload vulnerability.

The right ventricle (RV) exhibits a complex and distinctive geometry (Figure 1). It is composed of the inlet and outlet portions, separated by the crista supraventricularis and shares the interventricular septum with the left ventricle (LV), in addition to its own thin free wall that attaches to both the anterior and posterior septum [7]. The RV receives systemic venous return through the right atrium and propels deoxygenated blood into the pulmonary circulation. Despite generating a cardiac output comparable to that of the LV under physiological conditions, the RV operates against a much lower afterload owing to the low-resistance pulmonary vascular bed. This difference accounts for its thinner, more compliant wall structure compared to the LV [8]. The pulmonary circulation itself is a low-pressure, highly distensible circuit, characterized by a minimal increase in pulmonary arterial pressure even during states of markedly elevated cardiac output, such as exercise. This adaptation is largely due to the ability of the pulmonary vasculature to recruit and distend previously collapsed or underutilized vessels, as well as the intrinsically low basal vascular tone of the proximal pulmonary arteries. Unlike the systemic circulation, where muscular arterioles serve as resistors to modulate regional blood flow, the major pressure drop in the pulmonary circuit occurs at the level of the alveolar capillaries. Consequently, the transpulmonary gradient rarely exceeds 10 mmHg under resting physiological conditions [9]. The relatively low vasomotor tone of the pulmonary arteries also explains why acute pharmacologic modulation of pulmonary vascular resistance is limited compared with the systemic circulation. The structural design of the RV reflects its unique functional role. While the LV can be envisioned as a four-walled chamber with thick circumferential fibers generating concentric contraction, the RV is essentially a crescent shaped chamber wrapped around the LV. At the end diastole, the RV free wall is only 2–3 mm thick, in contrast to the 8–11 mm thickness of the LV free wall [10]. Although the RV produces less contractile force, its larger end-diastolic volume and surface-to-volume ratio enable it to achieve stroke volume similar to the LV but at a lower energetic cost [11]. During systole, longitudinal shortening predominates, with the apex moving toward the tricuspid annulus, a motion that accounts for the majority of RV stroke volume. The inflow and the outflow portions of the RV contract in a sequential pattern that facilitates forward flow into the pulmonary artery, while the high compliance of the pulmonary circulation allows ejection to extend into early diastole [12,13]. The differences between the ventricles translate into distinct hemodynamic responses. The LV, with its muscular wall, tolerates acute increases in afterload relatively well but is less suited to abrupt rises in preload. Conversely, the RV accommodates large fluctuations in venous return but responds poorly to sudden elevation in afterload [14,15]. As mean pulmonary artery pressures rises, RV stroke volume falls steeply and progressive RV dilation ensues, impairing contractility and promoting interventricular dependence. Because both ventricles share the septum, alterations in RV filling pressures directly affect LV geometry and function. Under physiological conditions, LV end-diastolic pressure exceeds that of the RV, allowing the septum to bow toward the RV. However, when RV end-diastolic pressure surpasses LV pressure, the septum shifts leftward, creating the characteristic “D-shaped” LV in cross sectional imaging [5,16]. This septal shift compromises LV filling and stroke volume and, in such settings, volume loading may paradoxically worsen cardiac output, as demonstrated in patients with pulmonary hypertension and chronic lung diseases [17]. Taken together, the unique anatomic configuration, contraction mechanics and coupling with the pulmonary circulation make the RV exquisitely adapted to handle variations in preload but highly vulnerable to sustained or abrupt increases in afterload. This structural-functional interplay explains why RV dysfunction represents a critical determinant of outcomes across a wide range of cardiopulmonary conditions.

### 2.2. Pathophysiology of Right Heart Failure: Acute vs. Chronic

Right-sided heart failure (RHF) arises from impaired right ventricular (RV) contractility secondary to pressure overload, volume overload or intrinsic myocardial dysfunction, culminating in a distinct clinical syndrome. The emergence of either subclinical RV dysfunction or overt RHF is a robust marker of adverse prognosis, irrespective of etiology. Although the unique embryologic origin of the RV and pulmonary circulation predisposes to dysfunction, the precise pathophysiological drivers remain incompletely elucidated. Recent investigative focus has turned to RV-specific inflammatory cytokines, pro-fibrotic mediators and metabolic alterations to unravel the molecular basis of RHF [18]. RHF may present in both acute and chronic forms. In either setting, increased afterload constitutes the primary adversary of the right ventricle. The fundamental distinction resides in the temporal dynamics of onset and the ventricle’s capacity for adaptation. Acute RHF represent a mechanical crisis characterized by abrupt hemodynamic collapse, whereas chronic RHF reflects a maladaptive process of progressive structural and functional remodeling (Table 1).

#### 2.2.1. Acute Right Heart Failure (ARHF)

ARHF is often precipitated by sudden increase in RV afterload or acute impairment of contractility rather than pure volume overload [4,19]. The thin-walled, compliant RV is exquisitely afterload sensitive and poorly tolerates abrupt pressure rises [5].

- Pressure overload

ARHF often arises from a sudden increase in RV afterload. Pulmonary embolism, acute pulmonary hypertension or severe hypoxic vasoconstriction can precipitate an abrupt pressure overload that exceeds the compensatory capacity of the RV. This sudden rise in afterload induces RV dilatation, wall stress and functional tricuspid regurgitation, with rapid progression to leftward septal shift, impaired LV filling, reduced cardiac output and systemic circulatory collapse. The magnitude of insult is determined not only by the mechanical obstruction or vasoconstriction itself, but also by the release of vasoactive mediators (e.g., thromboxane A2, serotonin), the presence of pre-existing cardiopulmonary disease and the limited ability of the RV to recruit contractile reserve under stress [20,21].

- Contractile dysfunction

Constitutes another critical mechanism. Acute RV myocardial infarction, typically secondary to proximal right coronary artery occlusion and concomitant with inferior infarction, results in ischemia-induced systolic impairment, diastolic stiffness and loss of synchrony. In this context, RV dilatation and impaired compliance exacerbate ventricular interdependence, restricting LV filling and compounding systemic hypoperfusion. RV involvement significantly worsens prognosis in inferior myocardial infarction [19,22]. Beyond ischemia, myocardial stunning after cardiopulmonary bypass, reperfusion injury or severe myocarditis can precipitate acute RV systolic failure. In each scenario, the transition from compensated RV dysfunction to frank ARHF is accelerated by the RV’s limited capacity for hypertrophy and metabolic adaptation under acute stress [23,24].

- Acute volume overload

Although better tolerated than pressure overload, volume overload can precipitate ARHF when severe or when superimposed on concomitant pressure or contractile insults. Acute severe tricuspid regurgitation, iatrogenic fluid overload or acute valvular disruption (e.g., chordal rupture) may provoke rapid RV dilatation, reduced ejection fraction and interventricular dependence [25].

Animal models of acute volume overload (e.g., via aorto-caval shunting, valve avulsion) demonstrate that RV tolerates volume better than pressure [26,27]. Isolated volume overload causes RV dilatation and reduced ejection fraction but seldom severe acute failure unless compounded pre-existing pressure overload, ischemia or other comorbidities. These models highlight that volume overload primarily exerts effects through ventricular interdependence and chamber enlargement, with a low solitary insult threshold for ARHF.

- Additional precipitants

Acute pericardial tamponade restricts RV filling through extrinsic compression, leading to precipitous preload dependence and hemodynamic collapse [28].

Tachyarrhythmias, particularly atrial fibrillation with rapid ventricular response or ventricular tachycardia, may abruptly reduce RV stroke volume, while bradyarrhythmias compromise forward flow and coronary perfusion [9]. Post-cardiotomy RV failure represents a specific syndrome driven by ischemia-reperfusion injury, inadequate myocardial protection or increased pulmonary vascular resistances (PVR) from pulmonary dysfunction [29].

Rare but catastrophic causes include acute aortic dissection involving the right coronary artery or trauma-induced disruption of RV free wall integrity. Moreover, acute pulmonary infections, ARDS and transfusion related acute lung injury (TRALI) can markedly elevate PVR, reproducing the pressure-overload paradigm [30].

- Molecular mechanism in ARHF

From a pathobiological perspective, ARHF reflects a convergence of mechanical stress, ischemia, inflammation and metabolic derangement.

Preclinical models of pressure induced ARHF reveal a rapid reversion to a fetal gene expression patter, characterized by upregulation of CC-and CXC-chemokines, stretch-sensing receptors and hypoxia inducible factors; downregulation of fatty acid transporters and mitochondrial oxidative enzymes; inflammatory activation with increased expression of MCP-1, neutrophil and monocyte chemoattractants (CINC-1, MIP-2) and infiltration of inflammatory cells leading to myocyte necrosis; metabolic dysregulation with suppression of key mitochondrial enzymes (NADH dehydrogenase, ATP synthase) and activation of protease like calpain, promoting cytoskeletal degradation and programmed cell death [31,32,33].

Models of volume overload also show altered signaling, including reduced phosphorylated ERK1/2 and dynamic changes in pathways involving TNF-alpha, TGF-beta1 and extracellular matrix (ECM) remodeling [34,35,36].

#### 2.2.2. Chronic Right Heart Failure (CRHF)

CRHF most commonly results from sustained RV pressure overload, particularly in pulmonary hypertension (PH) and secondary PH or from chronic volume overload, as observed in congenital and valvular disease [19,35]. The initial response to chronic pressure overload is typically adaptive remodeling, characterized by concentric RV hypertrophy, metabolic reprogramming with increased glucose utilization, angiogenic upregulation and controlled extracellular matrix deposition. These processes transiently normalize wall stress and preserve RV-pulmonary artery (PA) coupling, maintaining stroke work and cardiac output [37,38]. However, this compensatory phase is intrinsically limited. With persistent hemodynamic stress, the RV transitions to a maladaptive phenotype, marked by oxidative stress and inflammation, impaired angiogenesis due to inadequate capillary growth relative to hypertrophy, cardiomyocyte apoptosis, fibrosis and progressive ECM remodeling [19,39,40,41,42,43,44]. Clinically, this maladaptation manifests as decompensated RHF, with rising right atrial pressure (RAP), declining cardiac output and, in advanced stages, even falling pulmonary artery pressure (PAP) as a contractile failure dominates [45].

- Chronic RV volume overload

Long-standing volume overload, frequently encountered in adults with repaired congenital heart disease (e.g., tetralogy of Fallot, pulmonary atresia, hypoplastic left heart syndrome), severe tricuspid or pulmonary regurgitation and atrial septal defects, imposes a combined pressure and volume burden that predispose to CRHF [46,47]. Sustained abnormal loading drives progressive RV dilatation, annular enlargement with worsening functional tricuspid regurgitation (TR), hypertrophy, fibrosis and eventual systolic dysfunction. Pathophysiologically, volume overload induces mitochondrial dysfunction, oxidative stress, ECM remodeling and upregulation of natriuretic peptides and growth factors [48,49].

- Chronic RV pressure overload

Chronic RV pressure overload, as observed in pulmonary hypertension (PH), represents a pivotal determinant of morbidity and mortality, being a leading cause of right heart failure, lung transplantation and death [50]. Initially, the RV responds to sustained pressure overload through a spectrum of adaptative mechanism, including cardiomyocyte hypertrophy, metabolic reprogramming toward enhanced glucose utilization, angiogenic upregulation and controlled extracellular matrix deposition. These processes transiently preserve RV contractility and hemodynamic stability [37,38]. However, the adaptative phase is intrinsically limited and progressive pressure overload inexorably drives the transition to maladaptive remodeling characterized by RV dilation, systolic and diastolic dysfunction and ultimately overt RHF. Maladaptation is orchestrated by a complex interplay of cellular and molecular alterations. Impaired angiogenesis contributes to ischemia and cardiomyocyte apoptosis; fibrosis and inflammation increase myocardial stiffness and impair relaxation; metabolic dysregulation promotes a glycolytic shift, lipid imbalance, mitochondrial dysfunction and sustained neurohormonal activation, particularly via adrenergic and renin-angiotensin-aldosterone pathways, accelerates hypertrophy and fibrotic remodeling [41,42,43,44]. Experimental evidence also highlights the contribution of oxidative stress, endothelial dysfunction and genetic determinants (e-g., BMPR2 mutations) to the failure-prone RV phenotype [51,52]. Collectively, these maladaptive processes delineate a distinct pathophysiological trajectory of the RV under chronic pressure overload, mechanistically different from the left ventricle due to developmental, structural and metabolic peculiarities.

#### 2.2.3. RV Function in Pressure and Volume Overload

In chronic pressure overload, the RV initially undergoes adaptive concentric hypertrophy, maintaining stroke volume and contractile performance through increased wall thickness and preserved volumes. During this phase, exercise capacity and cardiac output may remain relatively intact. Once this homeometric adaptation fails, maladaptive remodeling ensues, characterized by eccentric hypertrophy, progressive dilatation and reliance on Frank-Starling mechanism to sustain forward output. This heterometric adaptation, however, comes at the cost of elevated filling pressures, increased wall stress and eventual clinical decompensation [53,54]. Functional decline is often heralded by dyssynchronous contraction, prolongation of RV systole beyond pulmonary valve closure and interventricular dyssynchrony, which impair ventricular-arterial coupling and exacerbate left ventricular underfilling [54,55,56]. Ultimately, RV systolic and diastolic dysfunction emerge, the latter driven by cardiomyocyte hypertrophy and interstitial fibrosis, further compromising compliance and filling [57]. In contrast, RV volume overload permits preservation of global contractility over prolonged periods, although contractile reserve is progressively diminished [58]. Ventricular interdependence plays a critical role: left ventricular dysfunction often arises not from impaired RV output, but from septal displacement, altered geometry and underfilling [59]. Disease-specific patterns of regional deformation also emerge. In atrial septal defect, apical hypercontractility compensates for volume overload [60], whereas in repaired tetralogy of Fallot, longitudinal strain is reduced, particularly in the free wall and apex, partly due to congenital myocardial architecture, chronic overload and surgical scarring [61,62]. Chronic volume overload, especially when compounded by pressure burden or marked chamber enlargement, ultimately predisposes to systolic failure and adverse outcomes. For this reason, timely corrective intervention is recommended before irreversible RV dilatation and dysfunction occur [19].

#### 2.2.4. From Chronic to Acute: The Continuum of Right Ventricular Failure

**Figure 2:**
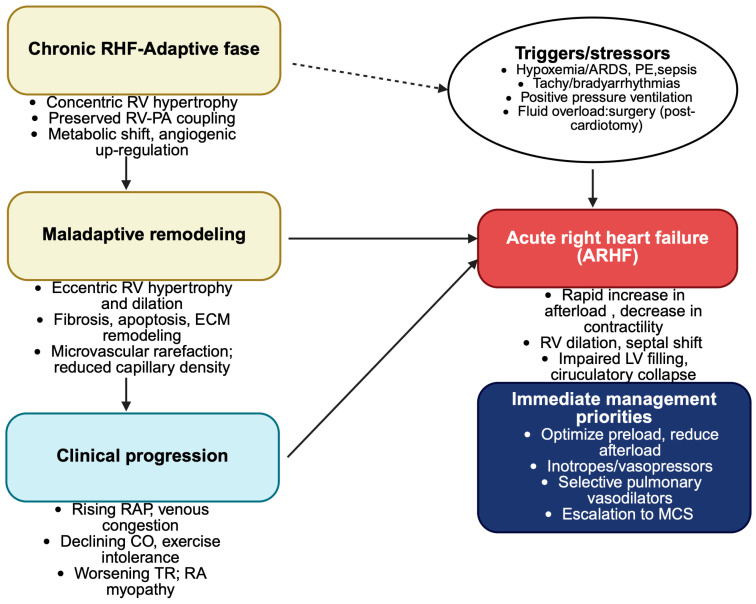
The transition from chronic remodeling to acute decompensation in right heart failure. Figure author-generated using BioRender.

Chronic right ventricular dysfunction, often secondary to congenital heart disease, arrhythmogenic right ventricular cardiomyopathy or long-standing valvular pathology represents a critical substrate for acute decompensation in the intensive care setting. Progressive remodeling, chamber dilatation and impaired contractile reserve gradually reduce the adaptive capacity of right ventricle, making it particularly vulnerable to acute hemodynamic stressors such as mechanical ventilation, sepsis, fluid overload or pulmonary embolism [63,64]. In patients with congenital heart disease, such as repaired tetralogy of Fallot, systemic right ventricle after atrial switch procedure, or Eisenmenger physiology, RV failure frequently develops insidiously but may precipitate abrupt deterioration when faced with the stressors typical if critical illness [65]. Similarly, arrhythmogenic disorders and chronic tricuspid regurgitation contribute to longstanding congestion and progressive RV impairment, which may suddenly destabilize under intensive care unit (ICU) conditions [66,67]. Recognizing this continuum between chronic and acute right heart failure is crucial, as the underlying pathophysiology shapes both the clinical presentation and the therapeutic response (Figure 2).

### 2.3. Clinical Manifestations of Right Heart Failure

The clinical phenotype of right heart failure reflects a combination of systemic venous congestion and impaired forward cardiac output. Congestive signs include jugular venous distension, hepatojugular reflux, peripheral edema, hepatosplenomegaly and ascites. In advanced stages, low-output manifestations emerge, such as hypotension, tachycardia, cool extremities, altered mental status and oliguria. The splanchnic compartment plays a pivotal role in disease progression: venous congestion and lymphatic overload disrupt intravascular fluid homeostasis, promoting interstitial edema, ascites and increased intra-abdominal pressure, thereby exacerbating cardiorenal interactions [25,68]. Biochemical abnormalities are common. Congestive hepatopathy and renal venous congestion result in elevated transaminases, impaired coagulation parameters and increased urea and creatinine [69]. Biomarkers of myocardial stress, including natriuretic peptides and troponin are frequently elevated, while systemic hypoperfusion may be reflected by increased lactate [70]. Anemia, present in up to one-third of patients, is typically multifactorial, involving iron deficiency and impaired responsiveness to erythropoietin [71].

### 2.4. Specific Etiologies and Management Strategies in RHF

RHF encompasses a heterogeneous spectrum of clinical scenarios, in which distinct etiological mechanisms dictate the trajectory of disease and therapeutic priorities (Table 2).

#### 2.4.1. RHF Secondary to Pulmonary Hypertension: Pre- vs. Post-Capillary Mechanism

RHF frequently develops as a consequence of increased RV afterload with pulmonary arterial hypertension (PAH) representing a paradigmatic condition. PAH represents a pre-capillary form of pulmonary hypertension, characterized by elevated mean pulmonary artery pressure and pulmonary vascular resistance with a normal or low pulmonary capillary wedge pressure (PCWP). In this setting, RV adaptation to chronic pressure overload is the key determinant of prognosis [72]. Therapeutic interventions therefore aim primarily at reducing RV afterload through modulation of pulmonary vascular tone and remodeling. Pharmacologic strategies in PH include several established classes: calcium channel blockers in vasoreactive patients at catheterization [73]; prostacyclin analogues (iloprost, Treprostinil, epoprostenol) improving symptoms, functional capacity and, in severe case survival [74]; phosphodiesterase-5-inhibitors (sildenafil, tadalafil) and endothelin receptor antagonist (bosentan, ambrisentan, macitentan) that enhance exercise capacity and hemodynamic parameters, with trials demonstrating reduced clinical worsening [75,76]; soluble guanylate cyclase stimulators (riociguat) and selective IP receptor agonist (selexipag), both of which have been shown to lower pulmonary vascular resistance and delay disease progression [77,78]. Beyond monotherapy, initial combination regimens, including dual or even triple therapy, are increasingly supported, particularly in high-risk patients, to achieve early and sustained hemodynamic and clinical improvement [79]. By contrast, RHF due to left-sided heart disease represents a post-capillary mechanism of pulmonary hypertension, driven by backward transmission of elevated left atrial and pulmonary venous pressures, leading to pulmonary venous hypertension, increased PCWP and RV dilatation. Management focuses on optimizing left-sided filling pressures and pulmonary congestion with directed heart failure therapies [80,81].

#### 2.4.2. Congenital Heart Disease-Associated RHF

Adults with congenital heart disease represent a growing population risk of PH and RHF [82]. In unrepaired lesions (atrial septal defects, ventricular septal defects, patent ductus arteriosus), chronic shunting promotes pulmonary vascular remodeling and eventual Eisenmenger physiology [83]. Once shunt reversal occurs, surgical closure is contraindicated and therapy becomes pharmacological. Evidence from controlled trials and registries supports the use of endothelin receptor antagonists, phosphodiesterase-5-inhibitors and prostacyclin analogues in these patients [84,85]. In repaired congenital lesions, particularly tetralogy of Fallot, long-term surveillance is crucial [86]. Residual pulmonary regurgitation, RV outflow tract obstruction and valvular dysfunction often lead to progressive RV dilatation and dysfunction, mandating serial imaging and timely intervention [87].

#### 2.4.3. RHF Secondary to Left Heart Disease

Left-sided heart disease remains the most common cause of RHF. Pulmonary venous hypertension, neurohormonal activation, coronary underperfusion of the RV and ventricular interdependence contribute to maladaptive RV remodeling. Prognosis is markedly influenced by RV function in both heart failure with reduced ejection fraction (HFrEF) and preserved ejection fraction (HFpEF) [88,89].

- In HFrEF, evidence-based LV therapies (RAAS blockade, beta-blockade, mineralocorticoids receptor antagonists, sacubril/valsartan, resynchronization therapy) are central, though their direct effects on RV performance remain incompletely defined. Preservation of euvolemia and reduction of pulmonary venous congestion are critical for RV protection [45,90].
- In HFpEF, RV dysfunction is highly prevalent and strongly prognostic [91]. Strategies aimed at reducing pulmonary vascular load have yielded mixed results: sildenafil trials were largely negative, whereas vericiguat showed promise in improving quality of life [92,93]. At present, no guideline-directed therapy is specifically recommended for RHF in the setting of HFpEF. In this patient population, meticulous volume management remains the cornerstone of treatment [87].

#### 2.4.4. RHF in Pulmonary and Thromboembolic Disease

Chronic lung disease and hypoxemia lead to cor pulmonale, where treatment hinges on optimization of the underlying respiratory condition and long-term oxygen therapy when indicated [90]. Chronic thromboembolic hypertension (CTEPH) represents another major etiology. Pulmonary endarterectomy remains the treatment of choice, with balloon pulmonary angioplasty offering a minimally invasive alternative for non-operable cases [94]. Riociguat has demonstrated efficacy in improving functional capacity and pulmonary vascular resistance in inoperable or residual CTEPH and is now guideline recommended [81,95].

#### 2.4.5. RHF in the Setting of LVAD Support

RHF complicates up to 40% of left ventricular assist device (LVAD) implantation, significantly worsening prognosis. Mechanisms include increased venous return, interventricular septal shift and preexisting pulmonary hypertension [96]. Management involves preoperative optimization with aggressive decongestion; intraoperative strategies to minimize transfusion and cardiopulmonary bypass times; postoperative treatment including inotropes (milrinone, dobutamine) and inhaled pulmonary vasodilators (nitric oxide, prostacyclin analogues, milrinone), which reduce pulmonary pressures and improve RV-arterial coupling; mechanical RV support in refractory cases [96,97,98,99].

#### 2.4.6. Acute Right Ventricular Ischemia

Acute right ventricular infarction, typically associated with proximal right coronary artery occlusion, leads to abrupt impairment of RV contractility. The clinical picture is often characterized by profound systemic venous congestion with relatively preserved pulmonary congestion. Early recognition and prompt revascularization are crucial, as the RV often exhibits a remarkable capacity for functional recovery once coronary perfusion is restored [100].

#### 2.4.7. Primary and Infiltrative Cardiomyopathies

Certain cardiomyopathies predominantly or disproportionately affect the right ventricle. Arrhythmogenic right ventricular cardiomyopathy (ARVC) is a genetic disorder characterized by fibro-fatty replacement of RV myocardium, predisposing to malignant arrhythmias and progressive RV dysfunction [101]. Infiltrative disease such as cardiac amyloidosis or sarcoidosis can involve the RV, leading to diastolic dysfunction and restrictive hemodynamics, frequently accompanied by biventricular failure [102,103].

#### 2.4.8. Right Sided Valvular Heart Disease

Severe tricuspid regurgitation, whether functional (secondary to RV or right atrial dilation, pulmonary hypertension or atrial fibrillation) or organic (degenerative, congenital or infective), constitutes an increasingly recognized contributor to chronic RHF. It leads to progressive systemic venous congestion, hepatic dysfunction and impaired forward cardiac output [104]. Less common but clinically relevant is pulmonary valve stenosis, most often of congenital origin, which induces chronic RV pressure overload and eventual failure if untreated [105].

#### 2.4.9. Pericardial Disease

Pericardial pathologies such as constrictive pericarditis and cardiac tamponade critically impair RV filling. In constriction, chronic pericardial thickening and fibrosis impose a rigid constraint on diastolic expansion, resulting in systemic venous hypertension and prominent RHF signs [106]. In tamponade, rapid accumulation of pericardial fluid leads to equalization of diastolic pressures and acute RV inflow obstruction, often culminating in hemodynamic collapse [107].

#### 2.4.10. Iatrogenic Causes

Certain chemotherapeutic agents, such as anthracyclines, can induce cardiotoxicity with biventricular involvement, sometimes predominating in the RV [108].

### 2.5. Assessment of Right Ventricular Function: Current Concepts and Advanced Imaging Approaches

**Figure 3:**
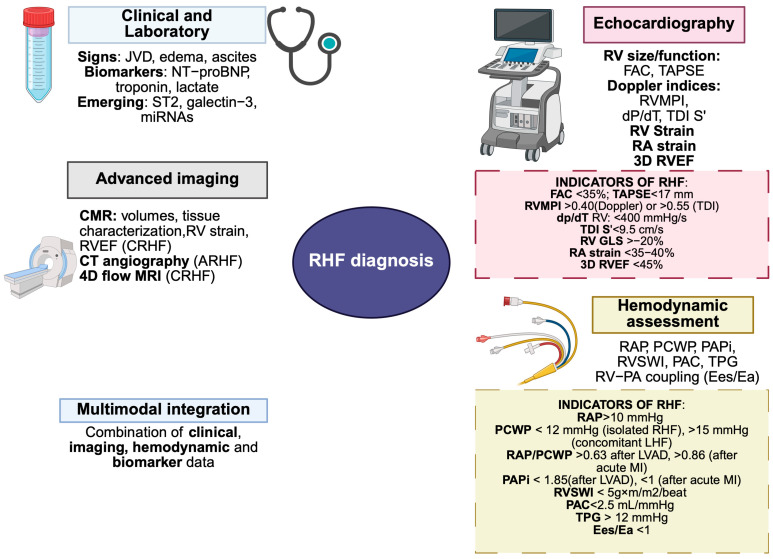
Diagnostic modalities in right heart failure. Figure author-generated using BioRender. RHF: right heart failure; JVD: jugular vein distension; FAC: fractional area change; TAPSE: tricuspid annular plane systolic excursion; RVMPI: right ventricular myocardial performance index; TDI: tissue doppler imaging; RV: right ventricle; RA: right atrium; GLS: global longitudinal strain; RVEF: right ventricular ejection fraction; CMR: cardiac magnetic resonance; CT: computed tomography; 4D flow MRI: four-dimensional flow magnetic resonance imaging; RAP: right atrial pressure; PCWP: pulmonary capillary wedge pressure; PAPi: pulmonary artery pulsatility index; RVSWI: right ventricular stroke work index; PAC: pulmonary arterial compliance; TPG: transpulmonary gradient; Ees: end-systolic elastance; Ea: pulmonary arterial elastance.

Assessment of RV function remains a complex and nuanced task, largely due to chamber’s unique crescentic geometry, thin-walled structure and longitudinal contraction predominance. These anatomical and functional peculiarities complicate accurate quantification of RV size and systolic performance. Importantly, RV function is highly load-dependent, with both acute and chronic variations in preload and afterload significantly influencing measured indices. Consequently, comprehensive evaluation typically necessitates a multimodality imaging strategy, complemented by hemodynamic assessment, to capture the integrated structural, functional and physiologic characteristics of the RV [109] (Figure 3).

Given the heterogeneity of RHF presentation, the diagnostic value of each modality may differ between acute and chronic settings (Table 3).

#### 2.5.1. Echocardiography

Transthoracic echocardiography (TTE) remains the most widely used modality for RV assessment due to its accessibility and ability to provide real-time functional information [110]. A structured evaluation should include:

- Dimensional analysis: 2D echocardiography enables measurement of basal, mid and longitudinal RV diameters, providing a prognostic information in conditions such as PH and acute pulmonary embolism (PE) [111,112].
- Systolic function indices: Fractional area change (FAC), derived from RV end-diastolic and end-systolic areas, serves as a validated surrogate for global RV systolic function (reduced FAC correlates independently with adverse outcomes, including mortality and major cardiovascular events) [113]; tricuspid annular plane systolic excursion (TAPSE), measured by M-mode, reflects longitudinal RV contractility (lower TAPSE values are predictive of increased mortality and urgent need for heart transplantation in chronic HFrEF patients) [114].
- Doppler-derived functional indices: RV myocardial performance index (RVMPI or Tei index) integrates systolic and diastolic performance [115]; dP/dT of the tricuspid valve and tissue Doppler imaging (TDI) of tricuspid annular systolic velocity (S’) provide additional prognostic insight, particularly in the setting of PH, RV infarction or post LVAD implantation [116].
- Right atrial function: although often overlooked, represents a crucial determinant of right sided hemodynamic and prognosis. The RA acts not merely as a passive reservoir but fulfills three complementary roles (reservoir during ventricular systole, conduit during early diastole and booster pump during atrial contraction). Impairment of any of these phases contributes to elevated right atrial pressure, systemic congestion and diminished RV filling. Advanced echocardiographic techniques, including speckle tracking-derived RA strain, have emerged as a sensitive indices of RA remodeling. Reduced RA reservoir and conduit strain are independently associated with adverse outcomes in PH, chronic heart failure and congenital heart diseas. Furthermore, atrial dysfunction frequently precedes overt RV failure and correlates with the severity of functional tricuspid regurgitation [117,118,119,120].
- Tricuspid regurgitation (TR) assessment: is a critical component of RV evaluation. Moderate-to-severe TR not only reflects annular dilatation and leaflet tethering secondary to RV enlargement, but also exacerbates systemic venous congestion and alters load dependent indices of RV performance. Severe TR can lead to underestimation of RV systolic pressure and confound the interpretation of echocardiographic parameters such as TAPSE or fractional area change. Moreover, TR severity has consistently been associated with worse prognosis, underscoring the importance of its systematic evaluation during RV functional assessment [121,122,123].
- Strain imaging: Speckle tracking echocardiography allows quantification of RV longitudinal strain which correlates with RV contractile reserve and has emerged as a sensitive predictor of adverse outcomes across multiple pathologies, including PH, chronic heart failure and congenital heart disease [124,125].
- 3D RV chamber quantification and 3D RVEF (<45% is considered the cutoff for RV dysfunction) [110,126]

#### 2.5.2. Advanced Imaging Modalities

While echocardiography offers a first-line assessment, other imaging techniques provide enhanced precision, tissue characterization and volumetric accuracy.

- Cardiac magnetic resonance (CMR) is the gold standard for RV volumetric quantification and ejection fraction assessment [109]. CMR allows accurate delineation of RV end-diastolic and end-systolic volumes, mass and RVEF, while also enabling myocardial tissue characterization for infiltrative or fibrotic disorders such as arrhythmogenic right ventricular cardiomyopathy (ARVC), cardiac sarcoidosis or amyloidosis. Late gadolinium enhancement (LGE) and T1/T2 mapping can provide further prognostic information regarding fibrotic burden and arrhythmogenic risk [127,128].
- 4D Flow MRI: Four-dimensional flow cardiac magnetic resonance has recently emerged as a powerful tool for the comprehensive assessment of right-sided hemodynamics. Beyond static volumetric data, it enables visualization and quantification of complex intracardiac flow patterns, vorticity and kinetic energy dissipation across the RV and pulmonary arteries. Although still primarily a research modality, 4D flow MRI offers unique mechanistic insights and holds potential for refining prognostication and therapeutic monitoring in RHF [129,130].
- Cardiac computed tomography (CT), particularly with contrast-enhanced angiography, is valuable in evaluating RV morphology in the context of pulmonary vascular pathology, such as acute PE or chronic thromboembolic pulmonary hypertension and in pre-surgical planning [131].

#### 2.5.3. Hemodynamic Assessment

Invasive measurements complement imaging by directly quantifying RV load and contractility [132].

- Right atrial pressure (RAP) to pulmonary capillary wedge pressure (PCWP) ratio is commonly used to characterize RV-pulmonary coupling, particularly after LVAD implantation or in acute myocardial infarction. The ratio is considered preserved when <0.6, whereas values ≥ 0.63 identify patients at increased risk of right ventricular disfunction in the setting of left ventricular assist device (LVAD) implantation [133,134].
- Pulmonary artery pulsatility index (PAPi), defined as (pulmonary artery systolic pressure (PASP)- pulmonary artery diastolic pressure (PADP))/RAP, is predictive of post-LVAD RV failure and overall prognosis in advanced heart failure. A PAPi < 1.5–2.0 is strongly associated with high RHF [134].
- RV stroke work index (RVSWI) quantifies RV contractile performance relative to afterload and reduced RVSWI is associated with increased risk of RV decompensation post LVAD or after acute RV infarction. Normal values typically range between 5–10 g×m/m^2^, whereas an RVSWI < 5 g×m/m^2^ indicates impaired RV contractility [135].
- Pulmonary arterial compliance (PAC), calculated as stroke volume divided by pulmonary pulse pressure (PAPP = pulmonary artery systolic pressure (PASP)-pulmonary artery diastolic pressure (PADP). PAC serves as a sensitive predictor of RV failure and adverse outcomes in chronic heart failure and PH, reflecting the dynamic interaction between the RV and pulmonary circulation. A PAC < 2 mL/mmHg indicates reduced RV-pulmonary coupling and portends a higher risk of progressive RHF [136,137].
- Transpulmonary gradient (TPG) serves as critical hemodynamic parameter for elucidating the etiology of right heart failure in the context of concomitant pulmonary hypertension. Hemodynamic profiling distinguishes between predominant pathophysiological mechanism: an elevated TPG (>12 mmHg) in conjunction with a normal PCWP (<15 mmHg) is indicative of a pre-capillary etiology, implicating pulmonary vascular disease. Conversely, a low TPG (<12 mmHg) with an elevated PCWP (>15 mmHg) signifies a post-capillary origin, typically due to left heart pathology. A scenario of both a high TPG and a high PCWP characterizes combined pre- and post-capillary pulmonary hypertension, reflecting the presence of overlapping vascular and cardiac dysfunction [138].

#### 2.5.4. Risk Stratification and Prognostic Score in RHF

Several predictive models have been developed to identify patients at risk of RHF following LVAD implantation, including the Michigan, STOP-RVF, CRITT and EUROMACS-RHF scores, which combine preoperative clinical, hemodynamic, echocardiographic and biochemical variables [139,140,141]. While these scores demonstrated good discrimination in their derivation cohorts, their performance in external validation has been less consistent, largely due to heterogeneous definitions of RHF, variability of population and the inability to incorporate intraoperative triggers or dynamic RV-LVAD dynamic interaction. Consequently, these tools remain useful for baseline risk stratification but should be interpreted with a broader clinical context. In addition to these composite models, simpler non-invasive surrogates have been proposed, such as the TAPSE/PASP ratio, where values < 0.31 mm/mmHg indicate RV-PA uncoupling and predict adverse outcomes [142,143]. Recent advances further emphasize the importance of RV-arterial coupling, defined by the ratio of RV end-systolic elastance to pulmonary arterial elastance (Ees/Ea). An optimal RV-PA coupling is typically maintained between 1.5 and 2. In compensated states, adaptive hypertrophy allows Ees to rise proportionally to Ea, preserving stroke work and efficiency. As maladaption progresses, the Ees/Ea ratio declines, heralding RV-PA uncoupling, reduced stroke volume and clinical decompensation. Non-invasive estimation via echocardiography or CMR is increasingly used for risk stratification and to guide therapeutic interventions [144,145]. Furthermore, multiparametric scoring systems integrating imaging, hemodynamic and biomarker data (e.g., NT-proBNP, troponin, lactate) offer superior prognostic accuracy compared to single parameter approaches [70].

#### 2.5.5. Emerging Biomarkers

Beyond natriuretic peptides and cardiac troponins, several novel biomarkers have been investigated as potential tools to refine risk stratification in RHF. Soluble ST2, reflecting myocardial stress and fibrosis, correlates with RV dilatation and adverse outcomes in pulmonary hypertension and advanced heart failure [146]. Galectin-3, a mediator of fibrogenesis and inflammation, has been associated with progressive RV dysfunction and systemic congestion [147]. Circulating microRNAs (miR-208, miR-126, miR-21) have shown promise as regulators of angiogenesis, apoptosis and ECM remodeling in experimental and clinical models of RV failure [148]. Additionally, markers of systemic inflammation and ECM turnover, such as growth differentiation factor-15 (GDF-15) and matrix metalloproteinases (MMPs), are increasingly recognized as contributors to the adverse remodeling phenotype [149]. Although still investigational, these biomarkers may complement imaging and hemodynamics by providing mechanistic insights and early signals of impeding decompensation. Beyond risk stratification, multiparametric assessment may also play a pivotal role in the early diagnosis of RHF, integrating subtle functional, hemodynamic and biomarker alterations that may not be captured by single parameter evaluation.

### 2.6. Therapeutic Strategies in Acute Right Heart Failure

**Figure 4:**
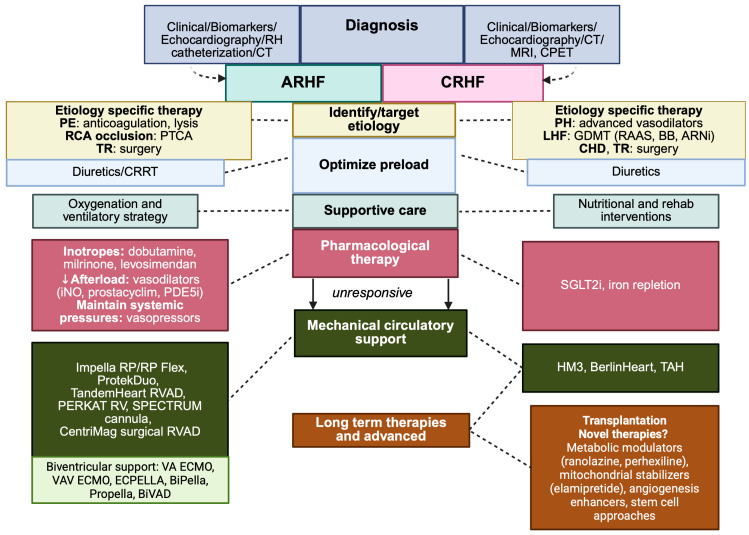
Stepwise therapeutic approach to right heart failure: acute and chronic presentations. Figure author-generated using BioRender. RV: right ventricle; iNO: inhalatory nitric oxide; PDE5i: phosphodiesterase type 5 inhibitors; ARHF: acute right heart failure; SGLT2i: sodium-glucose cotransporter-2 inhibitors; CRHF: chronic heart failure; PE: pulmonary embolism; GDMT: goal directed medical therapy; RAAS: renin-angiotensin-aldosterone system; BB: beta blockers; ARNi: angiotensin receptor-neprilysin inhibitor; CHD: congenital heart disease; TR: tricuspid regurgitation; VA ECMO: veno-arterial extracorporeal membrane oxygenation; VAV ECMO: veno-arterial-venous ECMO; RVAD: right ventricular assist device; Impella RP: Impella right percutaneous; BiVAD: biventricular assist device; TAH: total artificial heart.

The management of RHF must be tailored to the underlying etiology. Nevertheless, a comprehensive diagnostic assessment is essential in all patients, given the substantial overlap among therapeutic modalities (Figure 4).

#### 2.6.1. Medical Therapy

Medical management integrates pharmacological and supportive interventions aimed at optimizing preload, alleviating afterload and enhancing right ventricular contractility. Invasive hemodynamic monitoring with a Swan-Ganz catheter remains invaluable for dynamic assessment and titration of therapy [19].

#### 2.6.2. Targeting the Etiology

Defining the precipitating condition is critical to establish a condition-specific strategy: right ventricular infarction requires urgent reperfusion and cautious volume resuscitation; pulmonary embolism mandates anticoagulation with consideration of thrombolysis or percutaneous thrombectomy in selected patients; critical illness with systemic vasodilation necessitates meticulous volume handling, since excessive fluid administration in a vasodilated milieu can precipitate decompensation; in mechanically ventilated patients, minimizing inspiratory pressures is crucial to avoid iatrogenic RV afterload elevation.

#### 2.6.3. Preload Optimization

Although increased afterload is often the initial insult, therapeutic efforts usually begin with preload optimization, as this can be implemented promptly, monitored easily and produces rapid effects. The target is typically a central venous pressure of 8–12 mmHg [19]. Historically, the RV was considered a passive conduit, leading to the liberal use of volume loading. However, once RV dilatation develops, particularly in the presence of elevated afterload, wall stress rises steeply in this thin-walled chamber, causing further dilatation, tricuspid regurgitation, impaired LV filling, myocardial ischemia and multiorgan dysfunction [150]. Continuous infusion of loop diuretics, titrated to reduce right atrial pressure to the desired range, remains the most effective approach to offload the RV. In resistant cases, thiazide diuretics may potentiate natriuresis, while carbonic anhydrase inhibitors can mitigate alkalosis from combined diuretic use [151]. Renal replacement therapy (RRT) served as a rescue option for diuretic-refractory congestion, but should be instituted cautiously and is not considered first-line [19]. Conversely, in hypovolemic states such as RV infarction, cautious fluid replacement is required to restore filling without precipitating decompensation when afterload is elevated.

#### 2.6.4. Afterload Reduction

Therapeutic afterload reduction must be tailored to the specific pathophysiology. In the absence of a correctable trigger, pulmonary vasodilators are employed to reduce pulmonary vascular resistance. Intravenous milrinone exerts both pulmonary vasodilation and positive inotropy, but systemic vasodilation and hypotension often limit its use [152]. Inhaled agents, such as epoprostenol or nitric oxide, provide selective pulmonary vasodilation without systemic compromise. Oral phosphodiesterase-5-inhibitors have shown utility in specific settings (e.g., RHF following LVAD implantation) but must be avoided in postcapillary pulmonary hypertension due to the risk of pulmonary edema [4].

#### 2.6.5. Augmenting Contractility

Inotropes such as dobutamine and milrinone are most frequently used to enhance RV contractility and output. Milrinone, with potent vasodilatory properties and a renal clearance profile, necessitates careful dose adjustment in renal dysfunction. Dobutamine, with a shorter half-life and less pronounced vasodilatory effects may be advantageous in unstable patients. Levosimendan has also been shown to enhance RV systolic performance across several etiologies [152,153,154]. Vasopressors such as noradrenaline, while primarily alpha-adrenergic, exert modest beta1-mediated inotropy and may improve RV-pulmonary artery coupling when combined with inotropes [155].

### 2.7. Management of Chronic Right Ventricular Failure

Chronic RHF is approached with the same principles as the acute form but requires long-term strategies.

- Volume regulation: diuretics are central to reducing congestion and preventing RV volume overload, with close renal surveillance to avoid prerenal injury [156].
- Iron deficiency therapy: Iron deficiency is highly prevalent in chronic RHF, contributing to reduced exercise capacity, impaired skeletal muscle function and systemic inflammation. Intravenous iron repletion, particularly with ferric carboxymaltose, has been shown to improve functional capacity and quality of life in chronic left-sided HF, with emerging evidence suggesting parallel benefits in patients with right-sided dysfunction. Correction of iron deficiency not only alleviates anemia but also improves mitochondrial function and skeletal muscle energetics, potentially mitigating fatigue and exercise intolerance in RHF. Routine screening and treatment of iron deficiency should therefore be considered an integral component of long-term management [157].
- Afterload management: therapy depends on the underlying mechanism. In left-sided systolic dysfunction, evidence-based heart failure therapies (beta-blockers, ACE inhibitors, mineralocorticoid antagonists, ARNi and SGLT-2 inhibitors) are indicated. SGLT-2 (sodium-glucose cotransporter-2) inhibitors have transformed the management of left-sided heart failure, with consistent reduction in hospitalization and mortality across both HFrEF and HFpEF phenotypes. Although their direct effects on RV function remains less well studied, emerging data suggest potential benefits in reducing pulmonary pressures, improving systemic congestion and enhancing renal-cardiac interaction [158,159,160]. In pulmonary hypertension, treatment is disease-specific: group I PH require advanced vasodilator therapy (PDE5 inhibitors, prostacyclin analogues, endothelin receptor antagonist), whereas group IV disease necessitates lifelong anticoagulation with potential surgical endarterectomy due to chronic thromboembolic disease. Management of RHF in congenital heart disease requires specialist involvement due to complexity [4].

### 2.8. Mechanical Circulatory Support

**Figure 5:**
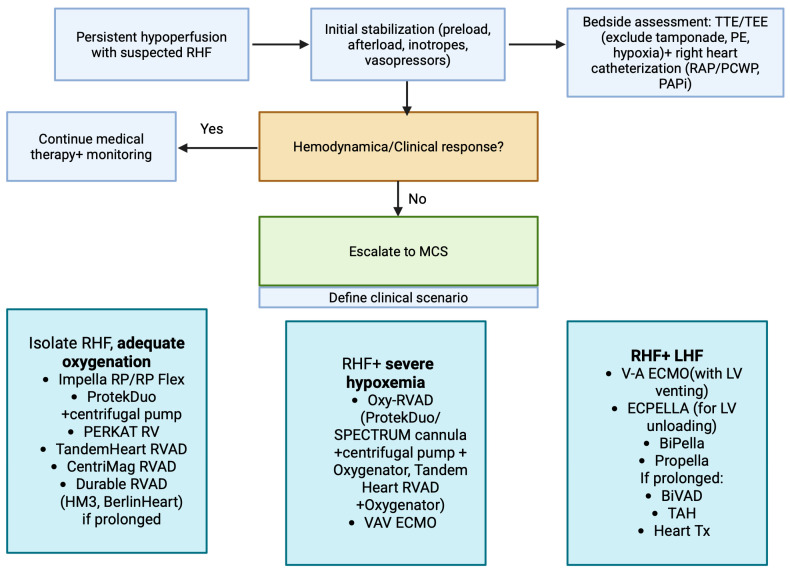
Decision algorithm for mechanical circulatory support. Figure author-generated using BioRender. TTE: transthoracic echocardiogram; TEE: transesophageal echocardiogram; PE: pulmonary embolism; RAP: right atrial pressure; PCWP: pulmonary capillary wedge pressure; PAPi: pulmonary artery pulsatility index; RVAD: right ventricular assist device; Impella RP: Impella right percutaneous; PERKAT RV: PERkutane KATheroumptechnologie RV; BiVAD: biventricular assist device; HM3: HeartMate3; Oxy RVD: RVAD with oxygenator; V-A ECMO: veno-arterial extracorporeal membrane oxygenation; RHF: right heart failure; BiVAD: biventricular VAD; Bipella: Impella RP combined with Impella CP/5.0/5.5; Propella: ProtekDuo with centrifugal pump combined with an Impella 5.0/5.5; TAH: total artificial heart; Tx: transplant.

In patients with refractory cardiogenic shock due to right ventricular failure unresponsive to maximal medical therapy, several mechanical circulatory supports (MCS) are available (Figure 5), (Table 4).

These devices may be employed either as a bridge to right ventricular recovery or as a bridge to definitive therapy, including heart transplantation [87]. Right-sided (MCS) system function by circumventing the dysfunctional right ventricle, either directly, diverting blood from the right atrium (RA) or right ventricle (RV) into the pulmonary artery (PA), or indirectly, by shunting venous return into the systemic circulation. In doing so, these devices increase LV preload, thereby augmenting cardiac output (CO) and improving systemic perfusion, while simultaneously relieving venous congestion [161]. Most devices rely on rotary pumps to generate forward flow. The delivered flow (Q) depends on both the pump’s rotational speed (RP) and the pressure gradient between the inflow and outflow sites, a parameter known as the pressure head (H). As H rises, Q decrease [161]. The majority of right-sided devices are intended for short-term support and are therefore used in intensive care setting. Attempts to develop durable, surgically implanted RV assist devices (RVADs) have not led to commercial availability. In practice, long-term RV support is occasionally achieved by repurposing left ventricular assist device (LVADs) in the right-sided circulation [162,163,164].

#### 2.8.1. Short Term Support

- Peripheral V-A ECMO is a RA to Aorta device: remains a widely used form of temporary biventricular support. Venous drainage from the RA is passed through a centrifugal pump and oxygenator, with return into the arterial system, typically via femoral circulation. Variants such as veno-arterialvenous (VAV) ECMO or veno-venous-arterial (VVA) ECMO have been developed to enhance oxygenation or RV unloading, respectively. Hemodynamically, V-A ECMO reduces RAP but has variable effects on PA pressures, depending on LV function and pulmonary vascular tone. In the presence of LV dysfunction, concomitant LV unloading via IABP or Impella (ECPELLA configuration) is often required. The left atrial veno-arterial (LAVA)-ECMO configuration, using trans-septal left atrial cannulation, allows simultaneous unloading of both ventricles and has the advantage of avoiding additional arterial access. However, this approach often provides greater circulatory support than required for isolated right heart failure. An alternative strategy is the use of a rotaflow centrifugal pump in a dedicated RVAD configuration, with venous outflow cannulated from the femoral vein or RA and return directed into the PA via a surgically implanted graft. The principal advantage of this system lies in its relative simplicity and low cost [165,166,167].
- ProtekDuo (LivaNova) is a single dual-lumen cannula inserted percutaneously via the internal jugular vein. One lumen drains blood from the right atrium, while the other lumen returns blood directly into the pulmonary artery. The blood drained from the RA flows through an external centrifugal pump that actively propels the blood into the PA, bypassing the failing RV. The pulmonary circulation is fully perfused ensuring oxygenation and reducing RV strain. The device decreases RAP and systemic venous congestion, increases CO and improves end organ perfusion [168].
- TandemHeart-RVAD (LivaNova) temporarily replaces RV function by actively moving blood from the right atrium to the pulmonary artery using a percutaneous centrifugal pump. The drainage right atrial cannula is usually inserted by femoral or jugular vein. The centrifugal pump provides continuous flow and bypasses the failing right ventricle, reducing RV workload. The pumped blood is returned to the pulmonary artery, usually via another percutaneous cannula, ensuring oxygenation of blood through the lungs while supporting the failing RV. The device reduces central venous pressure, improves CO and end-organ perfusion and provides a bridge to recovery, to decision or to longer term support [169].

Both TandemHeart-RVAD and ProtekDuo can incorporate an oxygenator (oxy-RVAD), allowing support in concomitant hypoxemic respiratory failure [170].

- Impella RP (Abiomed, Danvers, MA) is a single access, percutaneous micro-axial pump. Positioned via the femoral vein and advanced across the tricuspid and pulmonic valves into the PA, it withdraws blood from the RA and delivers up to 4 L/min into the pulmonary circulation. Unlike other systems, it cannot be combined with an oxygenator. Clinical data from RECOVER RIGHT trial demonstrated significant hemodynamic improvement, with reductions in right atrial pressure (RAP) and increases in cardiac index, with survival to discharge in approximately 73% of patients. The device has been successfully used in RHF complicating myocardial infarction, pulmonary embolism, LVAD implantation, post-cardiotomy shock and primary graft dysfunction after heart transplantation. Major limitations include femoral access, predisposing to bleeding, hemolysis and restricted patient mobility [171,172]. A new internal jugular configuration is under development to enable deambulation (Impella RP Flex, Abiomed) [173].
- Levitronix CentriMag RVAD (Abbott) is a surgically implanted extracorporeal centrifugal pump capable of flows up to 10 L/min. Unlike conventional pumps that rely on mechanical bearings, the CentriMag uses a fully magnetically levitated rotor, eliminating friction and reducing hemolysis and thrombosis. Cannulation typically involves sternotomy or thoracotomy with direct access to the RA/RV and PA. The pump withdraws blood from RA or RV through a venous cannula and propels it into the PA, thereby bypassing the failing right ventricle and ensuring adequate pulmonary circulation. While invasive, the device allows a rapid implantation and provides robust circulatory support. For these reasons it is frequently applied in post-cardiotomy shock, RHF following LVAD implantation and primary graft dysfunction after transplantation [174,175].
- PERkutane KATheroumptechnologie RV (PERKAT RV, NovaPump, Jena, Germany) is a percutaneous, pulsatile device designed to replicate physiologic RV ejection. Delivered via an 18F femoral catheter, it incorporates a nitinol stent-mounted balloon pump, electrocardiographically triggered to displace blood into the PA during diastolic inflation. Capable of generating flows up to 4 L/min, it has shown efficacy in preclinical models of RV failure, particularly in acute pulmonary embolism. Its key advantage is smaller bore access and pulsatile support, potentially mitigating microvascular dysfunction attributed to continuous flow devices [176,177].
- Spectrum medical dual lumen RV-PA cannula (Cheltenham, England) is a novel cannula placed via internal jugular vein, with inflow from the RA and RV and outflow into the PA. The outer lumen (which has multiple inflow openings) drains deoxygenated blood, while the inner lumen returns oxygenated blood back into the patient. Its dual-stage design addresses a key limitation of single-port RA drainage system (e.g., ProtekDuo), by capturing blood from both the RA and RV, thereby ensuring more complete RV unloading. Available in multiple sizes, it can provide 3–5 L/min of flow and is compatible with any extracorporeal centrifugal circuit with an oxygenator and allows preserved patient mobility [178].

#### 2.8.2. Long Term Support

- Durable RVAD (LVADs in RV position): dedicated durable RVADs are not commercially available. In cases requiring chronic RV support, LVADs may be repositioned to draw from RA or RV and eject into the PA. This off label strategy poses unique challenges: thinner RV/RA walls predispose to suction events, while low systemic pressures and high venous pressures complicate hemodynamics. Modifications, such as shortening the inflow cannula or restricting the outflow graft are often necessary. Debate continues regarding whether inflow is best positioned in the RA (less suction risk) or RV (more effective unloading) [161]. HeartMate 3 (HM3, Abbott): its use as a RVAD or as a part of biventricular support (BiVAD) is off label, though it has been done by implanting two HM3 pumps, one to support RV [179]. Berlin Heart (EXCOR) is a paracorporeal, pulsatile-flow VAD, configurable for support of LV, RV or both (BiVAD) and offers a long-term RV support, especially in pediatric cases, as part of a BiVAD strategy [180].
- Total Artificial heart (TAH) provide biventricular support (the blood is drained from the right and the left atria and is pumped by the TAH into the PA and aorta). The SynCardia TAH is pneumatically driven and generates pulsatile flow. In contrast, the Carmat Aeson bioprosthetic heart (another TAH) is electromechanically driven and incorporates biological valves and surfaces. Both systems can provide flows up to 9–10 L/min, ensuring full systemic and pulmonary perfusion [181,182,183].

#### 2.8.3. Special Consideration in Device Selection

- Tricuspid regurgitation (TR): TR is usually secondary to RV dysfunction with annular dilatation. While devices crossing the tricuspid valve may worsen or induce regurgitation, this should not preclude their use: TR may facilitate RV unloading, stabilize rotary-flow pump performance and, occasionally, improve with RV offloading [161,184].
- Concomitant LV dysfunction: In the presence of left ventricular dysfunction, the hemodynamic impact of right-sided devices must be carefully considered. Right- sided bypass systems (RA-PA or RV-PA) increase LV preload and may precipitate pulmonary edema in LV dysfunction, whereas RA-Ao configurations such as V-A ECMO elevate LV afterload, risking ventricular distension and pulmonary congestion. Under these circumstances, biventricular support provides a more balanced approach [161] (Table 5). Strategies such as BiPella (Impella RP combined with Impella CP/5.0/5.5), Propella (ProtekDuo and centrifugal pump combined with an Impella 5.0/5.5) or V-A ECMO with LV venting is preferred to achieve simultaneous unloading of both ventricles. Among these, BiPella offers the advantage of single arterial access, stepwise explantation and direct physiological decompression of the right and left ventricles, making it particularly attractive in selected patients with biventricular cardiogenic shock [185,186,187,188,189].

### 2.9. Heart Transplantation

Heart transplantation remains a last resort for refractory chronic RHF after all reversible causes have been excluded. Pure RV failure is an uncommon indication, typically limited to arrhythmogenic RV cardiomyopathy or extensive RV infarction. Outcomes are generally excellent, with 1-year survival near 90%. However, pre-transplant RVAD support, pulmonary hypertension and perioperative RV dysfunctions are associated with increased post-transplant mortality [19,190].

### 2.10. Novel Strategies for RHF

Recent advances in molecular cardiology have begun to elucidate the subcellular mechanism underlying RHF. Emerging evidence highlights distinct differences between the right and the left ventricle in energy metabolism, mitochondrial function, reactive oxygen species (ROS) handling, antioxidant defenses and angiogenic signaling, which may underlie their divergent responses to stress and offer novel therapeutic targets [191].

#### 2.10.1. Energy Metabolism Dysregulation

In pressure-overloaded RV, there is an early metabolic shift from free fatty acid oxidation to glycolysis, reducing oxygen demand for ATP production. While initially adaptive, progressive disease is characterized by diminished utilization of both glucose and fatty acids. Pharmacologic metabolic modulation has been explored in preclinical models of pulmonary hypertension and in clinical trials of LV failure with secondary RHF. Agents such as trimetazidine not only enhance glucose metabolism but also attenuate myocardial fibrosis via reduction of collagen deposition and connective tissue growth factor expression. Ranolazine, by inhibiting late sodium currents, mitigates electrophysiological and contractile dysfunction, decreasing myocardial oxygen consumption. Perhexiline has been reported to improve peak oxygen uptake, LV ejection fraction, exercise tolerance and skeletal-muscle energetics in small clinical cohorts [192,193,194].

#### 2.10.2. Mitochondrial Dysfunction, ROS and Antioxidant Capacity

Cardiac mitochondria are central to myocardial energy supply. RV mitochondria exhibit lower baseline membrane potential compared to LV; however, RV hypertrophy induces hyperpolarization. Inhibition of this hyperpolarization enhances contractility, suggesting a potential therapeutic avenue. Disruption of mitochondrial biogenesis has been implicated in decompensated RV failure, whereas biogenesis appears preserved in compensated states. Oxidative stress is amplified in the RV due to insufficiency early activation of antioxidant enzyme, predisposing to ROS-mediated damage and apoptosis [195,196]. Elamipretide, a mitochondria-targeted tetrapeptide, stabilized cardiolipin and improves electron transport chain efficiency, reducing ROS generation and promoting clearance, thereby restoring cellular bioenergetics in preclinical models [197].

#### 2.10.3. Impaired Angiogenesis

Chronic RV pressure overload is associated with defective angiogenic responses. Despite hypoxia-induced upregulation of HIF and VEGF signaling, RV hypertrophy exhibits uncoupling of these pathways, resulting in reduced capillary density, persistent ischemia and progressive fibrosis. Autopsy studies in HFpEF patients corroborate these findings, demonstrating decreased myocardial microvascular density and increased fibrosis. Experimental approaches aiming to enhance myocardial angiogenesis using targeted growth factors are under investigation, although the optimal combination and delivery strategies remain to be defined [198,199,200,201].

## 3. Discussion

RHF has long been regarded as a secondary or “consequential” phenomenon in the setting of left-sided heart disease. However, growing evidence emphasizes that RHF is a distinct and prognostically decisive syndrome, with unique anatomical, physiological and molecular underpinnings [19,202].

Epidemiological data underscore the magnitude of the problem. In patients admitted to intensive care units, acute right heart failure (ARHF) occurs in approximately 20–30% of those with cardiogenic shock and in up to 50% of patients with acute respiratory distress syndrome (ARDS) or severe pulmonary embolism [203,204]. In chronic disease, right ventricular is observed in nearly one-third of patients with left-sided heart failure with preserved ejection fraction (HFpEF) and carries a striking prognostic burden: 1-year mortality is nearly double in patients with RV dysfunction compared with those with preserved RV function. In pulmonary arterial hypertension, RV failure represents the leading cause of death, with 3-year survival rates dropping below 60% once overt RHF develops. These figures illustrate that RHF is not rare, nor benign, but a frequent and fatal complication across acute and chronic settings [19,63,91].

Anatomical and physiological considerations explain this vulnerability. The thin-walled, crescent-shaped RV is optimized for handling volume rather than pressure, functioning in close interplay with the pulmonary circulation. This evolutionary specialization, while efficient in normal physiology, translates into fragility under abrupt afterload stress. Acute conditions such as pulmonary embolism, septic pulmonary vasoconstriction and ARDS frequently precipitate right ventricular collapse, whereas chronic stressors like pulmonary hypertension or left-sided disease induce a more gradual maladaptive remodeling. The clinical implication is that RHF does not exist in a binary state but rather along a continuum of dysfunction, from compensated hypertrophy to overt decompensation [8,9].

From a pathophysiological standpoint, the dichotomy between acute RHF (ARHF) and chronic RHF (CRHF) remains essential, yet artificial if considered in isolation. Chronic remodeling creates a substrate of structural, metabolic and vascular changes that predispose to acute decompensation. Conversely, acute insults often accelerate chronic trajectories, reinforcing the concept of RHF as a dynamic process rather than a static entity. Cellular and molecular analyses provide a more detailed understanding of this framework. The RV exhibits earlier shift toward glycolysis under pressure overload compared to the LV, altered mitochondrial bioenergetics, increased oxidative stress in the absence of adequate antioxidant defenses and impaired angiogenesis. These maladaptive pathways contribute not only to functional decline but also to the limited efficacy of therapies directly transposed from LV failure [18,54,192,205,206].

Clinical manifestations of RHF remain protean and often underrecognized. Systemic venous congestion, renal and hepatic dysfunction, ascites and reduced exercise capacity are well documented, yet frequently overlap with or are masked by left-sided disease. Importantly, in critical care, acute RHF can complicate sepsis, acute respiratory failure or perioperative scenarios, leading to disproportionate hemodynamic instability. In this regard, RHF represents not just a cardiovascular disorder but a multisystemic syndrome, given its cascading effects on renal, hepatic, gastrointestinal and cerebral perfusion [19,81,90].

Diagnostic assessment has advanced considerably. Echocardiography remains the cornerstone, but novel modalities such as 3D echo, RV strain analysis and cardiac MRI have refined the ability to quantify systolic and diastolic function, interventricular interactions and remodeling. Invasive hemodynamics continue to provide unique insights in select settings, while emerging computational models and artificial intelligence promise to integrate multimodal data for patient-specific risk stratification. Such innovations may finally allow for earlier detection of subclinical dysfunction and tailored therapeutic interventions [4,207,208,209]. An essential component of RV functional assessment is tricuspid regurgitation. Moderate-to-severe TR not only mirrors RV dilatation and annular remodeling but also aggravates systemic congestion and alters load-dependent indices (e.g., FAC, TAPSE). In RHF, TR aggravates systemic congestion and independently predicts adverse outcomes, warranting its systematic inclusion in diagnostic and prognostic assessment [121,122,123,210].

Therapeutic strategies in RHF remain an evolving field. In the acute setting, treatment principles center around optimizing preload, reducing RV afterload and maintaining adequate perfusion with careful use of inotropes and vasodilators. In the chronic setting, pulmonary vasodilators, especially in pulmonary arterial hypertension, have revolutionized survival, though their applicability outside this field remains limited. Conventional therapies, such as diuretics, provide symptomatic relief but do not alter disease trajectory. Pharmacologic innovation, ranging from metabolic modulators (e.g., trimetazidine, ranolazine, perhexiline) to mitochondrial stabilizers such as elamipretide, offers suggestive potential, though clinical translation remains in its infancy.

A major frontier is mechanical circulatory support. Short-term devices (Impella RP, ProtekDuo, TandemHeart in RV configuration) have expanded the armamentarium in acute decompensation, whereas ECMO remains life-saving in refractory cases. Hybrid strategies, such as the BiPella and Propella approaches (combined LV and RV support) or V-A ECMO with LV unloading, reflect the recognition that RHF rarely occurs in isolation. In the chronic realm, durable LVADs in RV configuration, biventricular support and eventually total artificial hearts (TAH) provide bridges to transplantation or, in selected cases, destination therapy. Yet outcomes remain heterogeneous, with in-hospital mortality exceeding 40% in isolated RV-MCS implantation, emphasizing the need for better patient selection and device innovation.

Heart transplantation remains the ultimate option for end-stage biventricular failure. However, growing demand, donor scarcity and the increasing prevalence of patients with comorbidities challenge current allocation strategies. In this context, the ability to bridge patients effectively with MCS while preserving end-organ function is becoming increasingly critical [211,212].

Finally, emerging strategies point to a future where molecular and regenerative approaches may complement mechanical therapies. Gene therapies aimed at enhancing angiogenesis, stem-cell based interventions to restore RV microvasculature and novel pharmacologic targets for redox balance or metabolic reprogramming represent exciting frontiers. Their clinical translation, though still nascent, underscores a broader theme: RHF requires therapies tailored to its unique biology, rather than extrapolated from LV paradigms [213,214,215].

### Limitations and Strengths

As a narrative review, this work does not aim to provide a systematic or quantitative synthesis and selection bias cannot be entirely excluded. Moreover, the rapid evolution of diagnostic and therapeutic strategies may render some concepts subject to updates.

However, the review offers an integrative perspective that spans anatomy, pathophysiology, diagnostics and treatment, including mechanical support. This comprehensive framework bridges mechanistic insights with clinical practice and may serve as a reference for both clinicians and researchers.

## 4. Conclusions

RHF is increasingly recognized as a complex, multidimensional syndrome that extends beyond the “forgotten ventricle” paradigm. It encompasses acute critical illness and chronic cardiovascular pathology, with systemic repercussions that demand early recognition and nuanced management. Contemporary advances in imaging, molecular insights and mechanical circulatory support are progressively enabling tailored, physiology-driven interventions.

## 5. Future Directions

Future studies should refine prognostic markers and develop tailored therapies. Advances in imaging (4D flow CMR, speckle-tracking echocardiography), molecular biomarkers and multiparametric scores promote earlier detection and stratification. Although early data support the utility of 4D-flow CMR metrics (e.g., peak velocity, wall shear stress, vorticity) and advanced PET imaging for metabolic and innervation profiling, large multicenter, longitudinal studies are required to validate their prognostic role in RV function and RHF. Randomized trials are also needed to establish the optimal role of mechanical circulatory support and to test novel molecular targets, including metabolic modulation, mitochondrial protection and angiogenic stimulation. An integrative approach linking molecular insights with translational and clinical research will be crucial to improve outcomes in this high-risk population.

## Figures and Tables

**Table 1:** Comparison between acute and chronic RHF. PE: pulmonary embolism; ARDS: acute respiratory distress syndrome; RV: right ventricle; TR: tricuspid regurgitation; PH: pulmonary hypertension; CTEPH: chronic thromboembolic pulmonary hypertension; HF: heart failure; PR: pulmonary regurgitation; ASD: atrial septal defect; ARVC: arrhythmogenic right ventricular cardiomyopathy; PA: pulmonary artery; RAP: right atrial pressure; PAP: pulmonary arterial pressure; LV: left ventricle; CO: cardiac output; ECM: extracellular matrix.

	Acute Right Heart Failure (ARHF)	Chronic Right Heart Failure (CRHF)
Onset	Sudden, hours to days	Gradual, months to years
Predominant mechanisms	Abrupt pressure overload (massive PE, PH crisis, ARDS); Acute contractile dysfunction (RV infarction, myocarditis); Acute volume overload (severe TR, iatrogenic fluid overload); Mechanical compromise (tamponade, tension pneumothorax); Arrhythmic precipitants	Sustained pressure overload (PH, CTEPH, left-sided HF, chronic lung disease); Chronic volume overload (congenital heart disease, TR, PR, ASD); Progressive contractile dysfunction (ARVC, ischemia infiltrative disease); Constrictive pericarditis, systemic RV, pacing-induced dyssynchrony
RV remodeling	Acute dilatation, wall stress increase, impaired contractility	Adaptive hypertrophy, maladaptive dilatation, fibrosis, impaired RV-PA coupling
Hemodynamics	Rapid rise in RAP and PAP, reduced LV preload due to septal shift, low cardiac output, risk of circulatory collapse	Progressive increase in RAP, reduced CO, eventual fall in PAP in end-stage failure
Compensatory response	Minimal, overwhelmed within hours	Initially effective (hypertrophy, metabolic shift, angiogenic upregulation), later maladaptive (fibrosis, apoptosis, ECM remodeling, neurohormonal activation)
Clinical features	Acute hypotension, shock, syncope, acute systemic congestion, low output	Chronic venous congestion (edema, ascites, hepatomegaly), fatigue, exercise intolerance, cachexia
Extra-cardiac consequences	Acute renal/hepatic dysfunction due to low output and congestion	Congestive nephropathy, cardiac cirrhosis, gut edema, sarcopenia/cachexia
Prognosis	High in-hospital mortality if untreated (6–14%)	Progressive functional decline; morbidity and mortality depend on etiology (e.g., PH, systemic RV)

**Table 2:** Comprehensive etiologies of acute and chronic right heart failure, grouped into three principal mechanisms. RHF: right heart failure; PAH: pulmonary arterial hypertension; PH: pulmonary hypertension; ARDS: acute respiratory distress syndrome; CTPH: chronic thromboembolic pulmonary hypertension; COPD: chronic obstructive pulmonary disease; HF: heart failure; RCA: right coronary artery; HIV: human immunodeficiency virus; TRALI: transfusion related acute lung injury; TOF: tetralogy of Fallot; HLHS: hypoplastic left heart syndrome; AV: artero-venous; RV: right ventricle; ECMO: extracorporeal membrane oxygenation; AF: atrial fibrillation; RVR: rapid ventricular response; VT: ventricular tachycardia; CAD: coronary artery disease.

Mechanism	Acute RHF-Causes	Chronic RHF-Causes
Pressure overload	Acute pulmonary embolism (massive/submassive) Acute pulmonary hypertensive crisis (post-cardiotomy, post-transplant) Severe hypoxic pulmonary vasoconstriction (ARDS, pneumonia, asthma exacerbation) Acute pulmonary vasoconstriction from vasoactive mediators (thromboxane A2, serotonin) Acute aortic dissection involving RCA Sepsis with acute PH, TRALI	PAH (idiopathic, heritable, BMPR2 mutations) Secondary/post-capillary PH (left-sided HF, mitral/aortic valve disease) CTPH COPD, interstitial lung disease, sleep-disordered breathing, obesity, hypoventilation Chronic hypoxemia (high altitude, hepatopulmonary/portopulmonary hypertension, HIV-associated vasculopathy)
Volume overload	Acute severe tricuspid regurgitation (endocarditis, chordal rupture, device lead trauma) Acute pulmonary regurgitation (post-surgery, trauma) Iatrogenic acute fluid overload	Repaired congenital heart disease (TOF, pulmonary atresia, HLHS) Chronic tricuspid or pulmonary regurgitation Atrial septal defect, anomalous pulmonary venous return Systemic-to-pulmonary shunts High output states (large AV fistula, severe anemia, thyrotoxicosis) Chronic sequelae after valve replacement or congenital repair
Contractile dysfunction	Acute RV myocardial infarction (proximal RCA occlusion) Acute myocarditis Myocardial stunning (post-cardiotomy, ECMO weaning) Ischemia-reperfusion injury Acute pericardial tamponade or tension pneumothorax (mechanical compromise) Tachyarrhythmias (AF with RVR, VT) or severe bradyarrhythmias	Chronic ischemic RV disease (CAD, microvascular dysfunction) Arrhythmogenic RV cardiomyopathy Infiltrative cardiomyopathies (amyloidosis, sarcoidosis) Storage diseases (hemochromatosis) Chemotherapy/radiation-induced cardiotoxicity Chronic myocarditis Constrictive pericarditis, restrictive cardiomyopathy Systemic RV (congenitally corrected TGA, d-TGA after atrial switch) Chronic arrhythmias (AF with RA dilation, pacing-induced dyssynchrony, conduction delay)

**Table 3:** Comparative utilization of diagnostic modalities in acute versus chronic RHF (adapted from Konstam et al. [19] ARHF: acute right heart failure; CRHF: chronic right heart failure; TTE: transthoracic echocardiography; TAPSE: tricuspid annular plane systolic excursion; RV: right ventricle; FAC: fractional area change; TR: tricuspid regurgitation; RAP: right atrial pressure; CPET: cardiopulmonary exercise test; PE: pulmonary embolism; CT: computed tomography; US: ultrasound.

Diagnostic Modality	ARHF	CRHF	Comments
Clinical assessment	Critical for prompt identification of volume overload and hemodynamic compromise	Key for longitudinal monitoring of symptom progression and functional status	Remain cornerstone for diagnosis and follow-up
TTE	First-line imaging to assess RV size, function (TAPSE, FAC) and valvular competence	Serial monitoring of RV remodeling, function and TR severity	Non-invasive central tool in both setting
RH catheterization	Indicated in unstable patients or unclear etiology for precise hemodynamic guidance	Reserved for selected cases (PH severity, RAP, advanced therapy candidacy).	Invasive but pivotal in complex or refractory cases
CMR	Limited utility in acute setting due to logistical constraints	Gold standard for RV volumes, function and tissue characterization	High reproducibility and accuracy in chronic evaluation
Biomarkers	Adjunctive diagnosis and prognosis; useful for differential etiologies	Used for risk stratification and therapy monitoring	Integrates with clinical and imaging data refine diagnosis and prognosis
CPET	Rarely feasible in acute decompensation	Valuable for functional capacity assessment and elucidation exercise intolerance	Employed primarily for prognostication and therapeutic evaluation in stable chronic RHF
Pulmonary imaging (CT, lung-US)	Critical for exclusion or confirmation of acute causes such as PE	Not routinely used in CRHF management	Useful for differential diagnosis in acute settings

**Table 4:** Mechanical circulatory support (MCS) devices for right heart failure. ARHF: Acute right heart failure; LV: left ventricle; ECMO: extracorporeal membrane oxygenation; FF V-A ECMO: femoro-femoral venoarterial ECMO; LA: left atrium; RV: right ventricle; LV: left ventricle; RA: right atrium; PA: pulmonary artery; RVAD: right ventricular assist devices; PE: pulmonary embolism; RIJ: right internal jugular (vein); IJ: internal jugular (vein); MI: myocardial infarction; LVAD: left ventricular assist devices; PGD: primary graft dysfunction; TAH: total artificial heart.

Device	Access/Configuration	Flow (L/min)	Main Indication	Advantages	Limitation	Duration
V-A ECMO	Femoral/axillary veno-arterial circuit	>3–5	Refractory cardiopulmonary failure, ARHF with hypoxemia, biventricular shock	Full cardiopulmonary support, rapid deployment	LV distension, limb ischemia, bleeding, Harlequin syndrome (FF V-A ECMO)	Days-Weeks
TandemHeart RVAD	RA drainage with PA return (percutaneous)	3–5	Isolated RV failure, post-cardiotomy, post-LVAD, PE	Direct RV bypass, optional oxygenator	Invasive cannulation, bleeding, anticoagulation	Days-Weeks
ProtekDuo RVAD	Single site RIJ dual lumen cannula (RA to PA)	3–5	Isolated RV failure ± hypoxemia	Single venous access, allows ambulation	Cannula migration	Days-Weeks
Impella RP/RP Flex	Femoral (RP) or IJ (RP Flex), RA to PA	3–5	Acute isolated RV failure post MI, post-LVAD, post-surgery	Rapid deployment, no extracorporeal circuit	Hemolysis, valve trauma, short-term only	Days
CentriMag surgical RVAD	RA or RV to PA (surgical)	>10	Severe RV shock, post-LVAD, transplant PGD	High flows, stable support	Invasive, bleeding, infection	Days-weeks
Durable RVAD (off-label LVAD)	RA or RV inflow, PA outflow	>4–6	Chronic RV failure, BiVAD, bridge to transplant	Long-term support	Off-label, suction risk, surgical complexity	Weeks-months (potentially longer)

**Table 5:** Mechanical circulatory support (MCS) devices for biventricular support. BiPella: Impella CP/5.0/5.5 + Impella RP; LV Impella: left-sided Impella; CS: cardiogenic shock; RVAD: right ventricular assist device; LVAD: left ventricular assist device; V-A ECMO: veno-arterial extracorporeal membrane oxygenation; Propella: Impella 5.0/5.5+ ProtekDuo with centrifugal pump; ECPELLA: ECMO with Impella; LV: left ventricle; RV: right ventricle; BiVAD: biventricular assist device; TAH: total artificial heart; Tx: heart transplant.

Device	Access/Configuration	Flow (L/min)	Main Indication	Advantages	Limitations	Duration
BiPella	Impella 5.0/5.5 via axillary artery or Impella CP vie femoral artery + Impella RP via femoral/IJV	3–5 each	Acute biventricular CS	Minimally invasive, percutaneous, rapid deployment, stepwise explant possible	Emolysis, cost	Days-weeks
Propella	Impella 5.0/5.5 + ProtekDuo with centrifugal pump	3–5 each	Acute biventricular CS with hypoxemia	Single arterial + single venous access	Cannula migration, extracorporeal circuit required	Days-weeks
ECPELLA	V-A ECMO + Impella(for LV unloading)	>4–5	Refractory biventriculr shock	Full cardiopulmonary support, LV unloading reduces distension	Invasive, bleeding, limb ischemia, LV suction risk	Days-Weeks
Durable BiVAD	Dual LVAD (one in RV configuration)	4–6 each	Chronic biventricular failure, brige to Tx	Long-term support feasible, bridge to Tx	Off-label RV use, surgical complexity	Weeks-months (longer in selected)
BerlinHeart(EXCOR) in BiVAD configuration	Surgical paracorporeal pulsatile pumps	>7–10	Pediatric and adult chronic biventricular failure	Long-term support, bridge to Tx, pulsatile flow	Invasive, infection risk, cumbersome	Months/Years
TAH	Surgical biventricular replacement	>9–10	End-stage biventricular failure, bridge to transplant	Full systemic and pulmonary replacement	Size/device complications, specialized centers	Months (bridge to transplant)

## Data Availability

No new data were created or analyzed in this study. Data sharing is not applicable to this article.

## Abbreviations

The following abbreviations are used in this manuscript:

RHF	Right heart failure
RV	Right ventricular
ARHF	Acute right heart failure
CRHF	Chronic right heart failure
RVAD	Right ventricular assist devices
LVAD	Left ventricular assist device
BiVAD	Biventricular assist device
V-A ECMO	Veno-arterial extracorporeal membrane oxygenation
CHD	Congenital heart disease
TAH	Total artificial heart
BiPella	Left and right Impella support
Propella	ProtekDuo with centrifugal combined with Impella 5.0/5.5
